# What Is Meditation? Proposing an Empirically Derived Classification System

**DOI:** 10.3389/fpsyg.2019.02276

**Published:** 2019-10-15

**Authors:** Karin Matko, Peter Sedlmeier

**Affiliations:** Department of Psychology, Chemnitz University of Technology, Chemnitz, Germany

**Keywords:** meditation, classification system, diversity, embodiment, categorization, clusters, meditation techniques, multidimensional scaling

## Abstract

Meditation is an umbrella term, which subsumes a huge number of diverse practices. It is still unclear how these practices can be classified in a reasonable way. Earlier proposals have struggled to do justice to the diversity of meditation techniques. To help in solving this issue, we used a novel bottom-up procedure to develop a comprehensive classification system for meditation techniques. In previous studies, we reduced 309 initially identified techniques to the 20 most popular ones. In the present study, 100 experienced meditators were asked to rate the similarity of the selected 20 techniques. Using multidimensional scaling, we found two orthogonal dimensions along which meditation techniques could be classified: *activation* and *amount of body orientation*. These dimensions emphasize the role of embodied cognition in meditation. Within these two dimensions, seven main clusters emerged: mindful observation, body-centered meditation, visual concentration, contemplation, affect-centered meditation, mantra meditation, and meditation with movement. We conclude there is no “meditation” as such, but there are rather different groups of techniques that might exert diverse effects. These groups call into question the common division into “focused attention” and “open-monitoring” practices. We propose a new *embodied* classification system and encourage researchers to evaluate this classification system through comparative studies.

## Introduction

Meditation and mindfulness belong to two of the currently most popular and hyped research topics in psychology, psychiatry, medicine, and neuroscience (Van Dam et al., 2018). One issue that remains unsolved thus far, though, is providing a comprehensive theory of meditation (Sedlmeier et al., 2016). This is intensified by the fact that “meditation” is an umbrella term subsuming an extensive amount of diverse practices (Awasthi, 2013; Fox et al., 2016). Moreover, meditation is often used to describe both, the mental training technique employed by meditators as well as the resulting altered state of consciousness (Bond et al., 2009; Nash and Newberg, 2013). In this paper, we will mainly focus on the multiplicity of meditation *techniques*. To date, we have found no thorough overview of meditation techniques doing justice to the complexity and diversity of meditation practices found in various meditative traditions and schools. Additionally, there is no consensus on defining and demarcating meditation, left alone providing a truly encompassing classification system of meditation techniques. Our research aimed at filling this gap. By providing an copious list of commonly practiced meditation techniques (Matko et al., unpublished) and presenting the first empirically derived classification system of meditation in the present paper, we hope to contribute to an ongoing discussion and to an empirically grounded foundation for studying the effects of meditation, and, thus, working toward a future all-embracing theory of meditation.

Recently, researchers have begun to compare selected meditation techniques (Amihai and Kozhevnikov, 2014; May et al., 2014; Lumma et al., 2015; Fredrickson et al., 2017; Kropp and Sedlmeier, 2019; Trautwein et al., 2020) or proposed extended theoretical models and classification systems (Hölzel et al., 2011; Vago and Silbersweig, 2012; Nash and Newberg, 2013; Schmidt, 2014; Dahl et al., 2015; Lutz et al., 2015). Other researchers have elaborated on ancient theories of meditation tied to specific Hindu or Buddhist systems of thought (Lutz et al., 2007; Grabovac et al., 2011; Sedlmeier and Srinivas, 2016; Sedlmeier and Srinivas, in press). Whereas the latter approaches refer to specific combinations of meditation techniques, which are embedded in ethical and spiritual contexts, the former ones analyze and explain actually practiced meditation techniques, sometimes acknowledging but mostly bypassing ethical or spiritual context. Consequently, it seems hard to see through this variety of models and approaches, which reveal a lacking consensus among researchers.

Therefore, in an attempt to clarify and simplify these issues, we chose to develop a new classification system in a bottom-up way, relying on the judgments of experienced meditators. A similar approach has already been successfully employed in a study deducing a working definition of meditation by repeatedly asking a panel of seven experts in meditation research (Bond et al., 2009). In the present study, we requested a large sample of experienced meditators to compare a set of diverse, but commonly practiced meditation techniques according to the similarity of their expected general effects on practitioners. This approach was based on recent findings demonstrating how different meditation techniques yield different effects – phenomenologically, neuroscientifically, and psychologically. Hence, we concluded that this diversity of effects could provide a valuable means in detecting underlying dimensions that could help to structure the immense variety of meditation techniques. In the following, we will give a brief overview on the current state of the art.

Neuroscientific research has repeatedly shown differing brain activation and deactivation patterns during diverse meditative states following different meditative practices (Tomasino et al., 2014; Fox et al., 2016). Also, a growing body of research has found differential effects of specific types of meditation (Ospina et al., 2007; Sedlmeier et al., 2012, 2018; Goyal et al., 2014; Singer et al., 2016). So far, most research on the effects of meditation has been conducted without much theoretical foundation and has mainly been limited to four types of meditation: focused attention (mainly on the breath or the body), open monitoring, loving-kindness or compassion meditation, and mantra meditation (mostly Transcendental Meditation).

This limitation is probably due to the historical development of meditation research. While the 70’s saw considerable research efforts in examining the effects of Transcendental Meditation, the 90’s brought a huge interest into mindfulness meditation which keeps growing to the present (Schmidt, 2014). Meditation, though, is very diverse in itself, although it has been and is still largely being treated as a unitary construct. This is also reflected in the wide-spread use of “mindfulness meditation” as a synonym for meditation. A first attempt to differentiate meditation into “focused attention” (FA) and “open-monitoring” (OM) practices was brought forward by Lutz et al. (2008). Yet, as contemplative research kept expanding, more and more practices entered the field of interest. Whereas many models focus on describing the working mechanisms of mindfulness meditation (Grabovac et al., 2011; Hölzel et al., 2011; Tang et al., 2015; Grossenbacher and Quaglia, 2017), Vago and Silbersweig (2012) were among the first to propose diverse working mechanisms for three different styles of mindfulness meditation. These three styles of meditation are concentrative (FA), receptive (OM), and ethical enhancement (EE, e.g., loving-kindness or compassion) practices. Accordingly, now, “loving-kindness” and “compassion meditation” have been recognized as representatives of a new category as these practices entail elements of both, focused attention and open-monitoring styles of practice (Lippelt et al., 2014). More recent accounts have broadened the focus even further, as we will see in a moment.

Still, comparative studies have mainly focused on the above-mentioned four types of meditation, i.e., focused attention, open monitoring, loving kindness or compassion meditation, and mantra meditation. Studies empirically comparing some of these meditation techniques found differences in, e.g., dispositional mindfulness (Cebolla et al., 2017), creativity (Colzato et al., 2012), attention (Lee et al., 2012), affect (May et al., 2014), phenomenological experience (Przyrembel and Singer, 2018), or heartrate variability and perceived effort (Lumma et al., 2015). These differential effects highlight the possibility of categorizing meditation techniques into clusters of similar techniques according to their effects. Interestingly, another study (Amihai and Kozhevnikov, 2014) has shown that effects on the nervous system and cognitive performance depend more heavily on the tradition in which meditation is practiced than on the type of attentional system involved. Moreover, the framework in which certain meditation techniques are practiced seems to be of profound importance (Wachholtz and Pargament, 2005; Hunt et al., 2018; Trives, 2018). This rising complexity requires more complex models in explaining and classifying meditation.

Diverse meditation techniques have distinct effects. This finding has led to a few new proposals in classifying meditation including a greater diversity of meditation techniques from various backgrounds. Nash and Newberg (2013) chose to classify meditation practices into three domains of conjunct methods and states, i.e., cognitive, affective, and null domains. Another proposal (Dahl et al., 2015) categorizes meditation into three families, i.e., attentional, constructive, and deconstructive. Both proposals share certain commonalities. The cognitive domain and the attentional family both entail focused attention and open-monitoring practices. The affective domain and the constructive family both comprise techniques that aim at altering emotional responses, such as loving-kindness and compassion meditation. Dahl et al. (2015), however, expand the focus of this category and include Christian prayer and taking vows into their constructive family. The interpretation of the last category varies to some extent. While both approaches describe techniques and states aiming at dissolving the sense of self, closely related to “non-dual awareness” (Josipovic, 2010; Dahl et al., 2015) also include other “insight-oriented” practices, like the Buddhist Foundations of Mindfulness, Vipassana, Dzogchen, Koan, Mahamudra, and Muraqaba (a Sufi technique).

Yet, as reasonable as such considerations may be, these proposals have largely been derived top-down and, thus, might be limited by researchers’ personal expertise and experience. Consistent with this assumption, previous proposals omit several important and commonly practiced meditation techniques, especially from the Hindu context. Additionally, experienced meditators from the respective traditions might not agree with these classifications as the proposed clusters might be misguided attempts at throwing together “apples and oranges”.

Other approaches acknowledged these problems and devised phenomenological features or taxonomic keys to help researchers and practitioners describe what they are doing during meditation (Nash and Newberg, 2013; Schmidt, 2014; Lutz et al., 2015). Most taxonomies include questions on the object of meditation, the mental faculty or attentional mode employed, and the practical context of the practice (including questions on posture, guidance, setting, and breathing). They also touch on the effort, stability, and attitude or motivation behind the practice, or include questions on the axiological or traditional framework in which a practice is conducted. Furthermore, Lutz et al. (2015) emphasize cognitive and attentional factors, like degree of dereification (i.e., interpreting mental contents as mental processes rather than accurate depictions of reality), meta-awareness, and clarity. These taxonomies provide researchers and practitioners alike with valuable means of accurately describing their practice and allow to group or map meditation practices according to their phenomenology and contextuality. Still, the sheer number of taxonomies makes it difficult to detect commonalities amongst practices. Additionally, the diversity of meditation techniques makes it difficult to find an all-embracing definition of meditation and to properly differentiate meditation from other mind-body practices.

When people talk about meditation they often refer to someone sitting silently in a cross-legged position with closed eyes and searching for some inner silence or truth. This may be due to the historical development of meditation and meditation research in the West (see above) but cannot account for the immense variety of meditation practices found across different spiritual traditions. Interestingly, especially those approaches coming from our own Western Christian context, or from related Abrahamic traditions like Sufi mysticism and the Jewish Merkabah, have gotten less attention. Only now, there is an increasing interest into a more broad scientific exploration of meditation in its many forms, including rather atypical practices like Osho’s dynamic meditation (Bansal et al., 2016) or Kundalini Yoga (Peng et al., 2004). Yet, this broadening of the scope makes it even more difficult to find an overarching definition of what is actually meant by meditation. Some voices even discard the notion of finding a definition that “suits all” types of meditation (Schmidt, 2014). In the following, we will give some examples of the astonishing variety of definitions that have been brought up so far.

1. “The term *meditation* refers to a family of self-regulation practices that focus on training attention and awareness to bring mental processes under greater voluntary control and thereby foster general mental well-being and development and/or specific capacities such as calm, clarity, and concentration” (Walsh and Shapiro, 2006, p. 228).
2. “Meditation is an art of being serene and alert in the present moment, instead of constantly struggling to change or to become” (Deshmukh, 2006, p. 2239).
3. “Three main criteria have been defined as essential to any meditation practice: the use of a defined technique, logic relaxation (i.e., not intending to analyze, judge or expect), and a self-induced state/mode. Other criteria deemed important (by meditation experts) involve a state of psycho-physical relaxation, the use of a self-focus skill or anchor, the presence of a state of suspension of logical thought processes, a religious/spiritual/philosophical context, or a state of mental silence” (Bond et al., 2009, p. 135).
4. “A complex neural practice that induces changes in neurophysiology and neurochemistry of brain resulting in altered neurocognition and behavior in the practitioner” (Jaseja, 2009, p. 1).
5. “An exercise in which the individual turns attention or awareness to dwell upon a single object, concept, sound, image, or experience, with the intention of gaining greater spiritual or experiential and existential insight, or of achieving improved psychological well-being” (West, 2016, p. 4).
6. “A contemplative practice (e.g., meditation) is multi-generational, embedded in a community, has certain features which include an alteration of body and/or mind, and a certain kind of purpose or goal. There is a recipe (i.e., instructions) to achieve this goal, plus criteria for progress toward this goal which can be evaluated by evaluators from the community” (Dunne, 2018).

These six definitions are representative of opposing inclusion and exclusion criteria deemed important for labeling a practice meditation, or not. Some definitions are very specific (1, 3, 5), whereas others are rather broad (2, 4, 6). Specific definitions may be appropriate for investigating clear-cut research questions, they might, however, not account for the diversity of practices labeled “meditation”. Let’s consider the Sufi whirling technique (i.e., spinning around one’s own axis with arms spread out). This practice is generally considered a meditation technique aimed at “gaining greater spiritual or experiential insight” (5), it is thought to train awareness (1), and is a self-induced state of suspension of logical thought processes taking place within the context of a spiritual tradition (3). However, it does not necessarily involve a psycho-physiological relaxation (3), and does not necessarily focus on bringing mental processes under greater voluntary control (1), and might not be considered as dwelling upon a single object or experience (5).

On the other hand, very broad definitions could be very inclusive of diverse meditation techniques, but might broaden the scope too far by including practices generally not considered to be meditation techniques. This proves to be a tricky question, though. Can dancing, prayer, CrossFit or mindfully doing the dishes be considered meditation techniques or not? Where do we draw the line? According to definitions (2), (4), and (6) almost all the practices mentioned above would count as meditation. However, inducing “changes in neurophysiology and neurochemistry of brain resulting in altered neurocognition and behavior in the practitioner” (4) would also be true for experiences involving taking hallucinatory drugs or doing any kind of sports. As Ospina et al. (2007) point out, it might be “impossible to select components that might be considered universal or supplemental across practices” (p. 3). It might well be that the only thing all meditation practices have in common is the fact they are being called “meditation”.

This was the starting point for our research. We decided to approach the above-mentioned difficulties from a completely different perspective involving experienced meditators from diverse traditions in the process of gathering, selecting and grouping meditation techniques. We wanted to include as many different contexts, schools and traditions as possible. This bottom-up methodological approach differs fundamentally from any previous study, and stands in line with several researchers demanding a more rigorous investigation into the diversity of meditation techniques (Ospina et al., 2007; Travis and Shear, 2010). Our aim was to develop an empirical classification system of meditation techniques and to expand the scope of meditation practices under investigation. Thereby, we hope to inspire work toward developing one or more comprehensive theories of meditation.

As mentioned above, meditation techniques are often taught in a specific context, tradition or belief system. This context can influence the effects of meditation immensely (Amihai and Kozhevnikov, 2014; Hunt et al., 2018). Yet, little is known about the differential effects of specific meditation techniques, not to mention their interaction with context factors. For this reason, we chose to extrapolate basic meditation techniques common to many different contexts and traditions. This makes them easily accessible and understandable to many practitioners of diverse backgrounds and, thus, to researchers interested in the basic working mechanisms of meditation. Employing a bottom-up approach, we conducted two preparatory studies to extract diverse and recurring meditation techniques found in various spiritual traditions (Matko et al., unpublished). With this, we hope to provide a framework, which is independent of tradition-specific knowledge and easily applicable in any meditation (research) context.

Two preparatory studies were carried out to evaluate and deduce basic meditation techniques to be classified in the current study. In the first study (Matko et al., unpublished) we collected all meditation techniques we could find through literature search and by conducting guided interviews with 20 experienced meditators from diverse traditions such as Tibetan Buddhism, Theravada Buddhism, Zen Buddhism, Yoga, Hinduism, Tantra, Sri Chinmoy, Kundalini Yoga, Osho-Meditation, Christianity, Sufism, Brahma Kumaris and Qi Gong. Interviews focused on the central question “When you meditate, what exactly do you do?” The literature search included meditation manuals from different schools as well as research papers that included detailed descriptions of meditation practices (e.g., Dahl et al., 2015). This exhaustive search resulted in a list of 309 meditation practices that were reduced in several steps. Duplicates were removed, similar techniques were subsumed into one category, the level of abstraction was increased for techniques that were too specific, and techniques were excluded that were either too vague in their description (e.g., “catharsis”), or that were considered meditation practices of daily life rather than “formal practice” (e.g., mindfully doing the dishes). Technical terms (e.g., “chakra”) were replaced by more commonly used words (e.g., “energy center”) to make practices more easily comprehensible. This reduction led to a list of 50 basic meditation techniques.

The second preparatory study (Matko et al., unpublished) focused on exploring the prevalence, popularity, and comprehensiveness of our selection of 50 meditation techniques, and examined their underlying factor analytic structure. In a broad online survey, 637 experienced meditators from diverse meditation backgrounds provided data on how much experience they had with each of these 50 techniques. We found that all techniques were commonly used and could be grouped into seven factors. These factors represent groups of techniques that are commonly practiced together and correlate with the respective traditions they were derived from, but also span different traditions. In addition, we identified the 20 most popular meditation techniques widely practiced across many traditions (see section “Materials”, Table 1).

**TABLE 1:** List of 20 basic meditation techniques used in the present study, in their abbreviated and full descriptions.

**Abbreviated description**	**Full description**
Repeating syllables	Repeating syllables, words or phrases either mentally or loudly
Manipulating the breath	Voluntary manipulation of breath, e.g., reducing the strength of breathing or “pranayama” including holding one’s breath
Contemplating on question	Contemplating on a spiritually important question (e.g., “Who am I?”)
Walking and observing senses	Walking and being mindful of sensory perceptions (movement of the feet, legs, clothing, air, hair, etc.), coordinating it with the breath if necessary
Lying meditation	Lying down and going into a state of deep relaxation while being fully conscious
Concentrating on energy centers or channeling	Concentrating on one or subsequent locations in the body/“energy centers” (e.g., abdomen or Chakra, Dan Tien), including “channeling energy” through certain pathways (e.g., spine)
Observing the body	Observing how bodily sensations arise without adhering to them
Singing sutras or mantras	Singing sutras/mantras/invocations, alone or together with others
Contradiction or paradox	Concentrating the mind on something contradictory or complex without thinking discursively about the contradiction (e.g., Koan, Mahavakyas)
Body scan	Scanning the entire body (e.g., body scan), including perceiving and releasing occurring emotions and tensions
Concentrating on an object	Sustained concentration on an object or a visualized object (e.g., Kasina, geometrical pattern, picture of the master)
Meditation with movement	Carrying out predetermined, meditative sequences of movements
Sitting in silence	Sitting in silence (e.g., Shikantaza)
Observing thoughts or emotions	Observing how thoughts or emotions arise without adhering to them
Breath abdomen	Being mindful of the rise and fall of the abdomen while breathing
Opening up to blessings	Opening oneself up to blessings and inspiration
Meditation with sound	Meditation with sound (e.g., humming, or singing bowls)
Cultivating compassion	Cultivating compassion, sympathetic joy, equanimity, loving kindness^1^ (for oneself, friends, neutral people, enemies, the whole world), including Tonglen
Breath nose	Being mindful of the sensations arising in the nose during inhalation and exhalation
Visualizations	Visualization practices (e.g., heart as an opening rose blossom, body expanding in all directions, combining inhalation and exhalation with visualizations of energy, light, smoke, etc.)

*^1^These four features derived from Buddhist teachings which are called “brahmaviharas”. They refer to four “divine abidings” or “sublime states” which can be cultivated through meditation (Buswell and Lopez, 2014, p. 385). Cultivating these virtues, namely, loving kindness, compassion, empathetic joy, and equanimity, can help to counter “unwholesome states” like greed, ignorance and hatred, and may lead to living a more balanced life.*

This final selection of 20 techniques formed the basis of the current study, which focused on examining the structure underlying these diverse techniques. These structural investigations were based on intuitive similarity ratings of general effects that might be expected when meditators engage in practicing these meditation techniques. We decided to focus on general effects, because we were interested in the most general intuitions associated with these techniques. To make similarity judgments not too difficult (and to avoid activating textbook-knowledge about meditation), we did not further discriminate phenomenological, psychological or behavioral effects in our investigation.

We chose to pursue a purely statistical approach in this venture rather than referring to pre-existing concepts based on traditions or theoretical approaches. On the one hand, we were interested in identifying dimensions along which these 20 techniques could be classified and differentiated. On the other hand, we took an interest in potential clusters of similar meditation techniques that could be identified within these dimensions. The dimensions and clusters should form the basis of a new, empirically derived classification system for meditation techniques.

## Materials and methods

### Procedure

The current study used multidimensional scaling (MDS) as a tool for uncovering latent dimensions of diverse meditation techniques (Borg et al., 2018). Moreover, MDS techniques enable a researcher to produce a typology or, in our case, clusters of similar techniques using the judgments of a diverse set of individuals who are blind to the purpose of the study. Hence, MDS-based typologies are less prone to researchers’ biases than typologies developed through other methods (Robinson and Bennett, 1995). Accordingly, we employed MDS in our study for both means, detecting implicit dimensions and deriving clusters of similar techniques. The great advantage of the present method is that we can use the intuitive knowledge of the experienced meditators without additional rationalizations that might occur if we asked them directly about underlying dimensions for classifying meditation techniques.

The procedures followed in this study and the results are discussed below. The data were collected in accordance with ethical guidelines pertaining to the use of human subjects.

We devised an online survey to judge the 20 most popular meditation techniques identified in the two above-mentioned preliminary studies (see section “Materials”, Table 1) according to their similarity as perceived by experienced meditators. Participants saw the upper half of a matrix listing all 20 meditation techniques horizontally and vertically. They were asked to rate the similarity of each technique on a scale from 0 (no similarity at all) to 10 (very high similarity). The instruction read “Please indicate for each of the following meditation techniques how similar it is to each other technique regarding its general effects.” The instruction was phrased deliberately in a very general way and left open to participants’ interpretation because we were interested in detecting general structures and intuitive typologies associated with these 20 meditation techniques. If participants did not know a specific technique enough to rate it, they could enter –1 as a missing value. To control for potential sequencing effects, the order of presentation for the 20 meditation techniques was determined randomly for a first questionnaire. A second questionnaire was constructed with the order reversed. Each participant was randomly assigned one of the two questionnaires.

### Participants

The survey was sent to all participants of the second preparatory study mentioned above (Matko et al., unpublished) who had provided their e-mail addresses and consented to participate in further studies. A total of 102 experienced meditators completed the survey. Two participants were excluded because one did not provide sufficient data on his/her meditation routine, and one filled all answer fields with the same number. The final sample comprised 100 experienced meditators. The mean age was 52.90 years (*SD* = 10.78), the youngest participant being 26 years and the oldest 74 years old. 58.8% of the participants were female, and 93.0% were currently living in Germany.

Of all participants, 55.0% reported holding a university degree, 18.0% had completed higher education, 8.0% had completed their doctorate, and 11.0% had acquired a professional qualification. Regarding their occupation, 37.6% of participants were working as employees, 28.7% were self-employed, and 16.8% were retired.

#### Meditation Experience and Background

Participants had practiced meditation from 2 years up to 46 years, the mean meditation experience being 15.10 years (*SD* = 10.34). On average, they stated practicing meditation 6.97 times a week (*SD* = 5.48) for a mean of 31.90 min per session (*SD* = 22.60). 92.9% of all participants stated meditating regularly in the present, whereas 7.1% stated having meditated regularly in the past, but not in the present.

Participants were asked to select the tradition(s), the meditation techniques they practiced, were affiliated with. They reported practicing in the following traditions:

• Zen (*n* = 35)
• Theravada, Vipassana (*n* = 31)
• Tibetan Buddhism (*n* = 29)
• Yoga, Kundalini Yoga (*n* = 55)
• Osho meditation (*n* = 10)
• Other Hindu traditions, such as Vaishnavism, Sri Chinmoy, Sri Aurobindo, Mother Meera, Ramana Maharshi, Deepak Chopra, and Transcendental Meditation (*n* = 15)
• Mindfulness, MBSR (*n* = 31)
• Christianity (*n* = 10)
• Sufism (*n* = 6)
• Qigong, Tai Chi (*n* = 3)
• Judaism, Merkabah (*n* = 1)
• No Tradition, Free Meditation (*n* = 13)
• Other (*n* = 3)

Several participants had been practicing in different spiritual traditions, therefore, the total number of allocated traditions (*N* = 242) surpasses the total number of participants (*N* = 100). 33.7% of all participants practiced in only one tradition, 48.5% practiced in two or three traditions, and the remaining 17.8% practiced in more than three traditions. These traditions could belong to a similar background, e.g., diverse Buddhist traditions, or could differ in their backgrounds.

### Materials

Participants were asked to rate the similarity of the 20 basic meditation techniques listed below. Table 1 displays the full description of each technique (as it was used in the survey) as well as the abbreviations utilized in the following text.

## Results

**FIGURE 1:**
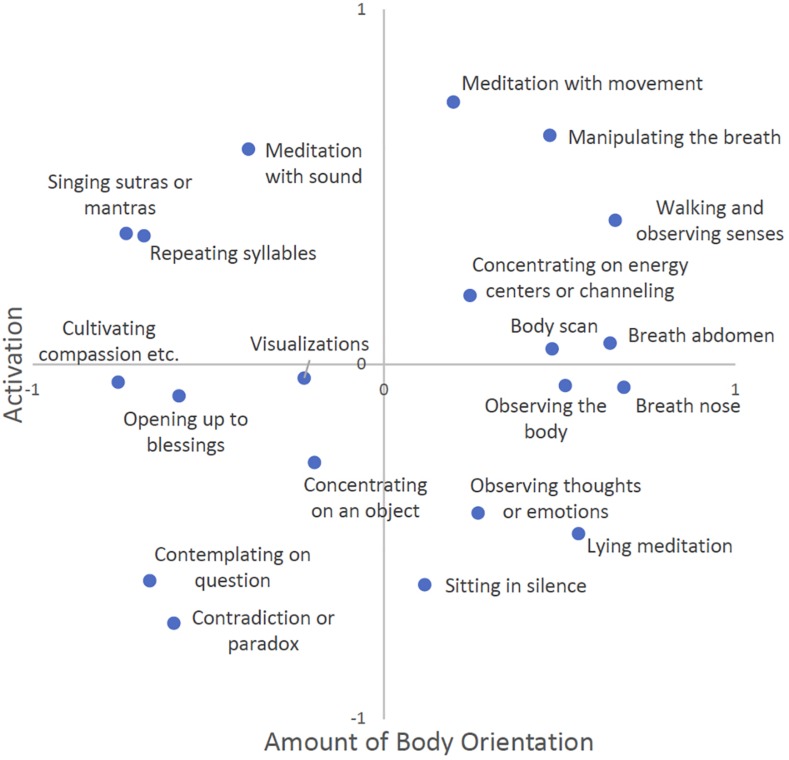
Overall multidimensional scaling (MDS) solution, based on average similarity judgments of *N* = 100 experienced meditators.

Multidimensional scaling can be applied to any similarity matrix. Thus, each participant’s ratings could serve as the basis for analysis. However, as an individual’s ratings can be very subjective or limited to his or her personal experience, we calculated the means of the similarity judgments across all participants. On the basis of the resulting similarity matrix, we performed a multidimensional scaling analysis using SPSS’s program PROXSCAL. Following recommendations by Borg et al. (2018, p. 78), we applied a multiple random starts configuration (*n* = 1000) and a stress convergence of 0.00001. Model fit was measured using the stress value, a measure depicting the aggregated representation errors of each data-distance pair (Borg et al., 2018, p. 29). Kruskal’s stress values for the one-, two-, and three-dimensional models were 0.207, 0.043, and 0.020, respectively. Adding the second and third dimensions reduced stress (by 0.164 and 0.023, respectively), suggesting an increasingly better fit with the data. The improvement from the two- to the three-dimensional model was negligible, though. Therefore, we opted for the two-dimensional model (Figure 1).

The statistical method of multidimensional scaling returns a dimensional output with potentially meaningful clusters, which are open to interpretation by the researcher. Thus, we thoroughly inspected Figure 1 and arrived at the following interpretation described below.

Dimension 1 has cultivating compassion and other brahmaviharas (see footnote 1), contemplating on a spiritual question, concentrating on a contradiction or paradox, and singing sutras or mantras on one extreme. At the other end are scanning the body, observing the abdomen or the nostrils while breathing, lying meditation, and manipulating the breath. Meditation techniques with a more abstract or conceptual focus received lower scores, while those with a higher amount of body-orientation received higher scores. Therefore, we decided to label this dimension “amount of body orientation”.

Dimension 2 has sitting in silence, concentrating on a contradiction or paradox, lying meditation, and observing thoughts or emotions at one end of the spectrum, while meditation with movement, walking and observing senses, and manipulating the breath are at the other end. Lower scores reflect a more passive, still and contemplative kind of meditation practice, while higher scores reflect more (bodily) active kinds of meditation practices. This dimension was labeled “activation”.

**FIGURE 2:**
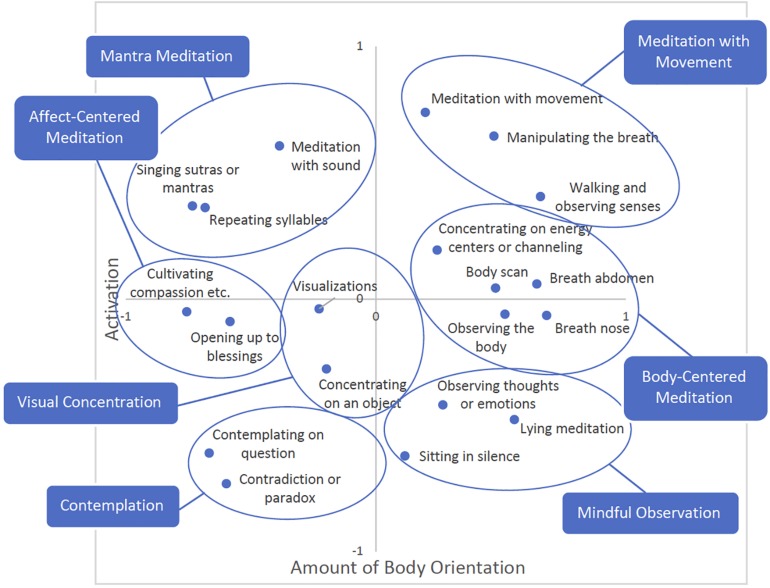
Overall multidimensional scaling (MDS) solution with indicated clusters and labels.

We identified seven clusters of meditation techniques by visual inspection (Figure 2). The biggest cluster includes five techniques that have a strong focus on observing the body, the breath or sensory perceptions, and was labeled “body-centered techniques” (middle right). It also includes concentrating on locations in the body or “energy centers”. Another cluster comprises practices that focus on mindfully observing oneself in stillness and was, thus, labeled “mindful observation”. It includes lying meditation, sitting in silence, and observing thoughts and emotions. These meditation techniques are relatively close to so-called “open-monitoring” techniques (see respective descriptions in Table 1). Manipulating the breath, walking and observing senses, and meditation with movement could be grouped into a broader cluster of “meditation with movement”.

Four smaller clusters were identified on the other side of the diagram, toward the more conceptual or object-oriented end. One cluster comprises concentrating on a contradiction or paradox and contemplating on a spiritual question and was labeled “contemplation”. The second cluster includes visualizations, and concentrating on an object or a visualized object, and was labeled “visual concentration”. The third cluster comprises opening oneself up to blessings, and cultivating compassion, loving-kindness, sympathetic joy, or equanimity, and can be seen as a cluster of “affect-centered techniques”. The forth of the smaller clusters includes repeating syllables, words or phrases, singing sutras or mantras, and meditation with sound, and was labeled “mantra meditation”.

These two dimensions, plus the seven clusters of meditation techniques, constitute our proposed new classification system of meditation techniques.

### Differences Between Traditions

Results from one of the preliminary studies (Matko et al., unpublished) identified differences in usage and popularity of certain meditation techniques between meditators of two major traditions, i.e., Buddhist and Hindu meditators. Therefore, we decided to conduct another two multidimensional scaling analyses for these two subgroups. We wanted to see whether meditators from these two traditions have different conceptions about clusters and dimensions of basic meditation techniques.

Participants who stated practicing in one or more Buddhist traditions (i.e., Zen, Theravada, Vipassana, Mindfulness, or MBSR), but in no other tradition from another spiritual background, were allocated to the group of Buddhist meditators (*n* = 27). Participants practicing in one or more Hindu traditions (i.e., Yoga, Kundalini Yoga, Hindu traditions, or Osho), but in no other spiritual tradition, formed the group of Hindu meditators (*n* = 20). Participants of any other spiritual or mixed spiritual backgrounds were excluded from the following analyses.

**FIGURE 3:**
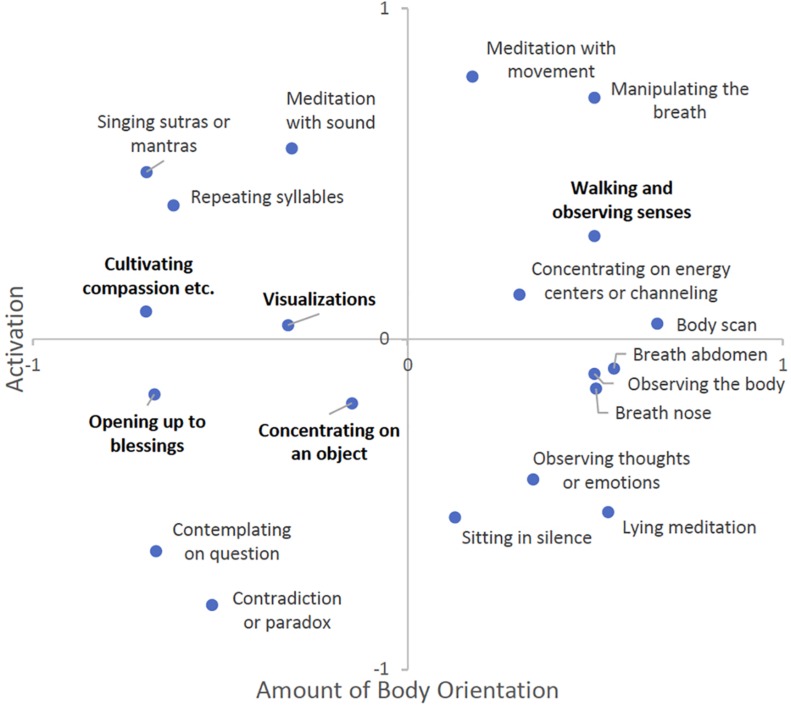
Multidimensional scaling (MDS) solution for Buddhist meditators (*n* = 27).

**FIGURE 4:**
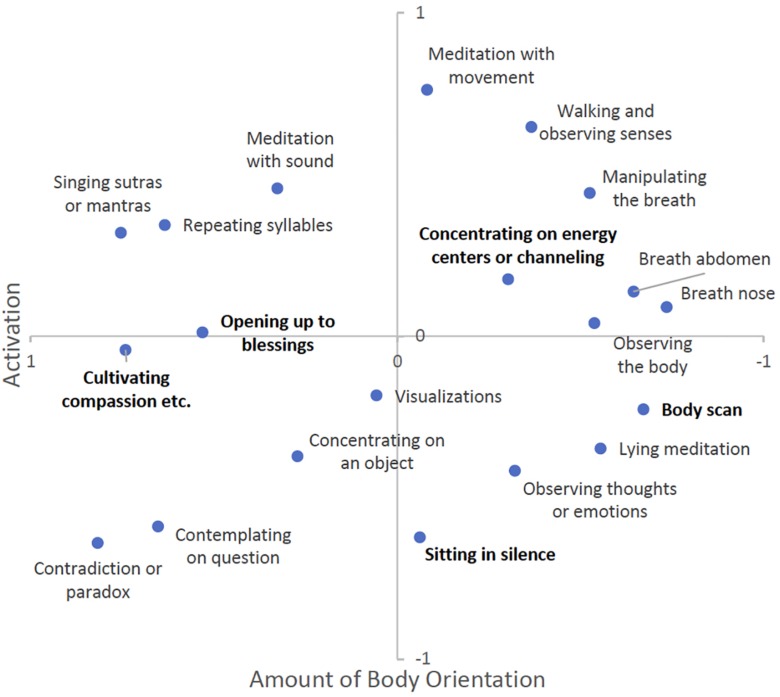
Multidimensional scaling (MDS) solution for Hindu meditators (*n* = 20).

In accordance with the results mentioned above, we selected two-dimensional models with Kruskal’s stress values of 0.054 (Buddhist), and 0.048 (Hindu), respectively. These values indicate an adequately good, but not perfect fit between model and data. Figure 3 shows the solution for Buddhist meditators, and Figure 4 for Hindu meditators (the original solution was mirrored along the *X*-axis to increase comparability between solutions), respectively.

First, both figures indicate a high congruence between ratings. Both solutions, Buddhist and Hindu, bear similarity not only to each other, but also to the overall solution described above. Moreover, both solutions exhibit similar dimensions, namely “amount of body orientation” as Dimension 1, and “activation” as Dimension 2, and similar clusters of techniques. However, and importantly, some of the techniques have slightly shifted their position and/or affinity to the afore-mentioned clusters (printed in bold type).

The solution for Buddhist meditators shows basically the same cluster structure as the overall solution, with two small peculiarities. First, the technique walking and observing senses shifted from the “meditation with movement” to the “body-centered meditation” cluster. This appears reasonable when looking at Buddhist traditional practices where walking meditation is commonly practiced alternating with breathing meditation (Kornfield, 2009; Sedlmeier, 2016). Second, the distances increased between opening up to blessings and cultivating compassion, and visualizations and concentrating on an object, respectively. This may be indicative of an assumedly greater differentiation of these techniques in Buddhist meditators.

Looking at the solution of Hindu meditators, both dimensions can be replicated, again. Looking closer, certain shifts in location lead to changes in some clusters. Contrary to the Buddhist solution, opening oneself up to blessings and cultivating compassion remain in the same cluster. However, both techniques moved further away from the “visual concentration” cluster, and now appear to be closer to the cluster of “mantra meditation”. Second, the body scan has left the cluster of “body-centered techniques” and joined lying meditation and observing thoughts and emotions. This appears reasonable with regard to Hindu practice traditions, especially in the Yoga context where the body scan is commonly practiced in a supine position (Ott, 2013). Third, two techniques appear to form categories of their own as they are distant to and cannot be grouped with any of the other clusters. These two techniques are “sitting in silence” and “concentrating on energy centers or channeling”.

All in all, there is remarkable conformance between the original solution and both solutions from Buddhist and Hindu meditators, respectively. At the same time, both solutions reveal tradition-specific particularities that can easily be attributed to traditional frameworks and modes of practice. This gives further evidence to the general validity of the proposed classification system.

## Discussion

The present study arrives at a novel classification system for meditation techniques and seems to be the first attempt to have been devised in an empirical and systematic bottom-up way. Drawing upon results from extensive preparatory studies (Matko et al., unpublished), we employed multidimensional scaling (MDS) to the similarity ratings of 100 experienced meditators for 20 well-known and diverse meditation techniques. Thus, our approach is unique not only in its methodology, but also in the variety of meditation techniques that were included in the analyses.

During the past years, the scope of scientific research has expanded continually to include a wider range of meditation practices (Nash and Newberg, 2013; Dahl et al., 2015). However, this expansion has made it even more difficult to find an overarching definition or top-down classification that could account for the diversity found in meditation practices (Schmidt, 2014). Therefore, we chose to rely on the judgments of experienced meditators, a method that has already been successfully employed by other researchers in the field trying to find demarcation criteria for meditation (Bond et al., 2009). This approach has the advantage of getting close-up practice-based insights from practitioners from a variety of spiritual backgrounds.

Based on our results, we propose a two-dimensional system of classifying meditation according to (1) the amount of body orientation in the technique, and (2) the level of activation in the technique. Furthermore, we propose seven main clusters of meditation techniques, namely: (1) Body-centered meditation, (2) mindful observation, (3) contemplation, (4) mantra meditation, (5) visual concentration, (6) affect-centered meditation, and (7) meditation with movement.

Taking a closer look at the dimensions and clusters, implicit assumptions of experienced meditators differ fundamentally from afore-mentioned theoretical proposals. Former proposals have focused on cognitive variables to describe and differentiate meditation techniques with the popular “focused attention” (FA) and “open monitoring” (OM) distinction (Lutz et al., 2008) leading the way. But also newer models (Nash and Newberg, 2013; Dahl et al., 2015) have attributed differences in techniques largely to diverse cognitive mechanisms or attentional modes at work. Thereby, the involvement of different cognitive mechanisms should result in differential effects. For instance, meditation practices from the attentional family (Dahl et al., 2015) should increase attention and decrease mind-wandering, whereas meditation practices from the constructive family might affect the regulation of emotion.

### Meditation Is Inherently Embodied

Quite on the contrary, our MDS solution does not depict cognitive, but rather embodied dimensions. Both dimensions, amount of body orientation and level of activation, are closely related to bodily processes. They have been taken into account in previous descriptive or taxonomic systems (Nash and Newberg, 2013; Schmidt, 2014), but have not been considered as central dimensions, yet.

It seems that, according to experienced meditators, meditation is inherently somatic. This is in line with research on “embodied cognition” (Damasio, 2006; Varela et al., 2017). Damasio (2006, 2012) convincingly argues that past and present states of the body heavily influence the contents and processes in the brain and that body-based emotions and feelings shape our mind. Nowadays, there is growing consensus among researchers that cognition is shaped by both, top-down (descending pathways from the cerebral cortex) and bottom-up (ascending pathways from the periphery) processes (Clark, 1999; Thompson and Varela, 2001; Wilson, 2002; Barsalou, 2010; Winkielman et al., 2015). This view has also been recently discussed in the context of contemplative science and mindfulness research (Michalak et al., 2012; Kerr et al., 2013; Cebolla et al., 2016; Khoury et al., 2017).

Meditation places the focus of attention onto those ascending and descending inner processes, making them more salient in consciousness. Many, if not all, meditation practices emphasize directing attention to interoceptive and/or exteroceptive signals. Whether meditators observe all incoming (sensory or mental) stimuli, develop compassionate feelings for others, silently repeat a mantra, or explicitly focus on bodily mechanisms such as the breath: the body remains a constant companion in all their endeavors. Likewise, Buddhist teachings emphasize that body and mind are equally valid objects of meditation, and encourage practitioners to use awareness of the whole body as a somatic anchor for mindfulness, or, in other words, cultivate an embodied form of mindfulness (Anālayo, 2016). Thus, it appears shortsighted to define meditation based on purely cognitive or attentional dimensions. We suggest that all meditation techniques have a somatic component and meditation is inherently embodied. We ground these assumptions in the results of our empirical analyses.

It is surprising, though, that meditators implicitly classify meditation techniques on embodied dimensions, although we had requested them to judge the similarity of general effects of these techniques. We do not know whether participants judged techniques according to similar phenomenological experiences likely to occur during the practice of the specific technique or according to similar psychological outcomes following the prolonged practice of the technique. It might well be that they considered the one or the other, or both, or something completely different. But, we can probably assume that these diverse interpretations of the question were leveled out by agglomerating the judgments of 100 participants. Overall, it seems that similarity or dissimilarity of meditation techniques seems to be implicitly attributed to differences in the two above-mentioned embodied dimensions, i.e., level of activation and amount of body orientation.

Yet, one issue that should be looked at more closely in further research is whether similarity on these two dimensions corresponds to differences in actual effects, too. Future studies should evaluate whether meditation techniques judged high in one dimension, e.g., level of activation, lead to similar phenomenological experiences, neuroscientific signatures, and/or psychological and behavioral outcomes. They could also compare techniques judged high versus techniques judged low in one dimension and investigate whether there is a linear increase or decrease of specific effects along this dimension. A close-up examination of diverse techniques could reveal similar or dissimilar mechanisms and processes underlying the practice of these techniques and take into account individual variation between participants.

### Expanding Focused Attention and Open Monitoring

The commonly made distinction between FA and OM practices could not be replicated in the present study, confirming previously voiced considerations (Amihai and Kozhevnikov, 2014). Also, looking at Buddhist practices, the same meditation objects can serve for concentrative (shamata) as well as insight (vipassana) meditation techniques (e.g., Pa Auk, 2003). Moreover, some commonly used techniques, such as the body scan might be arguably seen as a mixture of (sequentially moving) focused attention and open awareness. So, it might not come as such a surprise that both, OM and FA practices are displayed in our model in a far more differentiated way.

Still, it is easier to connect the cluster of “mindful observation” techniques with OM practices than to make connections to the FA concept. The mindful observation cluster includes three main practices in which the present-moment experience is approached with an open and receptive attitude (Bishop et al., 2004). These are sitting in silence, observing thoughts and emotions, and lying meditation. In contrast, FA practices are possibly the most difficult to locate in our model. Many studies used breathing meditation as a form of FA or “concentrative meditation” (Valentine and Sweet, 1999; Manna et al., 2010; Ainsworth et al., 2013; May et al., 2014). Mantra meditation has also often been considered a specific form of FA (Fox et al., 2016, p. 211). On the contrary, some researchers have emphasized a different mode (“automatic self-transcending”) being supposedly active in mantra meditation (Travis and Shear, 2010). In this form of practice, the repetition of the mantra becomes successively more subliminal until it fades away into silence. Looking at our MDS solution, breathing meditation and mantra meditation are very distant from each other, underlining differences in presumed effects and mechanisms at work in these two techniques. Therefore, it does not seem plausible to subsume them into the same category of FA meditation.

Furthermore, observing the breath might be considered concentrative for novice meditators, but might progress into a more passive and observational mode with growing experience, or even change back and forth from concentrative to receptive during the same meditation session. This would also be true for other practices clustered together in our solution as “body-centered meditation,” i.e., scanning or observing the body, and concentrating on a location in the body or on “energy centers”. The only identifiable cluster of techniques with a strong concentrative focus is the group of “visual concentration” techniques. Conversely, these have a very specific visual focus (concentrating on an object such as a picture or disk, or visualization techniques). Thus, they can be considered to be a very specific form of FA.

In conclusion, we would suggest that it might eventually be helpful to discard the rather unspecific category of FA meditation and replace it by the more specific categories presented in our empirically derived MDS solution. This notion needs further empirical investigation, though. Future experimental intervention studies should evaluate whether the alleged differences between clusters can be replicated in empirical findings. According to our model, meditation techniques that are closer to each other in the MDS solution should yield more similar effects than techniques which are further apart from each other.

We encourage researchers to evaluate this new classification system through comparative empirical studies. These studies could investigate short-term and long-term effects of each technique or cluster of techniques taking into account phenomenological, psychological, behavioral and neuroscientific aspects. They could also try to capture individual experiences with and reactions to these techniques. Single-case research designs (Barlow et al., 2009) seem to be a promising approach in capturing differential effects of diverse meditation techniques. They enable custom-tailored measurement and high-resolution recording of specific changes over time, also regarding individual differences in responses to meditation (Sedlmeier et al., 2016). Several authors have pointed out that individual differences might tremendously influence the effects of meditation (Hölzel et al., 2011; Lippelt et al., 2014; May et al., 2014). Also, the phenomenological experiences encountered during meditation depend heavily on individual factors, such as personality or learning history (Schmidt, 2014). This individual variation might be of particular interest in the context of investigating diverse meditation techniques, especially regarding person-technique-fit. Consequently, future studies should include measures of personality traits and carefully capture individual responses to different forms of meditation.

### Limitations and Future Directions

We are well aware that our choice of practices might have been limited to the regional availability of meditation teachers and traditions. Still, the comprehensiveness of our selection was confirmed by a broad sample of experienced meditators in one of the preparatory studies (Matko et al., unpublished). This allowed us to deliberately choose the 20 most popular and commonly known techniques for the current study. Additionally, the MDS solutions of ratings of primarily Buddhist or Hindu meditators were astonishingly congruent with the overall solution, while, at the same time, revealing tradition-specific particularities. This shows how different traditional background knowledge can frame responses, even though the question we asked our participants was very general in nature. We might have received different answers if our question had been more specific, e.g., specifying phenomenological, psychological or behavioral effects. Yet, this was not the aim of the present study, which rather focused on detecting general structures and intuitive typologies. Future studies could evaluate more specific typologies of meditation.

One could argue that the 20 techniques, which were investigated in this study, were artificially constructed and taken out of context. Traditionally, meditation techniques are practiced in a specific order, in the framework of a specific tradition, or in combination with other practices. Observing the breath, e.g., is often combined with visualizations or with the repetition of a mantra. Conversely, little is known about the effects of combined meditation techniques compared to simple techniques. Therefore, it seems promising to investigate and compare both, simple and combined techniques, and see if there are, indeed, any additive or interactive effects.

The same is true for the sequence of practices and the specific framework or traditional context of practice. To date, there is a scarcity of studies into sequence or framework effects (Hölzel et al., 2011; Lippelt et al., 2014). Nevertheless, some studies have shown a greater perceived effort for participants who began their practice with loving-kindness meditation compared to beginning practice with breathing meditation or the body scan (Lumma et al., 2015; Kropp and Sedlmeier, 2019). Additionally, a specific traditional background or framework can tremendously influence the effects of meditation (Wachholtz and Pargament, 2005; Amihai and Kozhevnikov, 2014). However, these effects may be very complex, specifically regarding the manifold meditation traditions. For this reason, and as a first step of investigation, we chose to take out meditation techniques of their traditional context and evaluate their basic effects. This might help to effectively disentangle genuine effects of simple meditation techniques from the effects of their traditional context. Future studies could compare the effects of bare meditation techniques to a combined intervention of meditation practice and ethical or philosophical teachings.

Another argument could be that some of the 20 meditation techniques employed in this study are not simple techniques but already rather broad categories in themselves. For example, voluntarily manipulation of breath, visualizations, or meditation with movement are all conglomerates subsuming a lot of diverse techniques. This was our conscious choice in the interest of brevity. During our preparatory studies we came across a great variety of techniques (*N* = 309, list available on request) which had to be considerably shortened for the current study, for pragmatic reasons. In this way, we ensured that participants’ load during the similarity ratings remained manageable. At the same time, it allowed for comparisons to be fine-grained enough for our analysis, incorporating a great variety of meditation techniques. The remaining variety surpasses by far the range of meditation techniques examined in earlier studies.

Nonetheless, these broad categories of techniques should be eventually investigated in greater detail. For instance, manipulating the breath, also often referred to as “pranayama,” can take many forms. These can range from reducing the strength of breathing, to alternate nostril breathing with holding one’s breath, or even very rapid breathing (Ott, 2013; Ott and Epe, 2018). Investigations into different forms of “pranayama” or breathing techniques have already shown significant and differentiated effects on cardiovascular variables and stress (Peng et al., 2004; Conrad et al., 2007; Sengupta, 2012; Bhavanani et al., 2014). Still, some traditions regard breathing techniques as preparatory exercises rather than meditation techniques. For this reason, it would be interesting to further compare the effects of diverse breathing techniques, and, successively, compare them to other basic meditation techniques.

The other two broad categories mentioned above can be very diverse, too. Visualizations can focus on imagining light or fire at different body parts, imagining the body expanding in all directions, or merging with a visual representation of a deity (a typical Tibetan Buddhist practice, see, e.g., Amihai and Kozhevnikov, 2014). Meditation with movement includes techniques of Yoga, Qigong, Tai Chi, Osho meditation and other movement-based meditation traditions. These traditions are incredibly rich in the variety of techniques they offer (Ospina et al., 2007). Similar to sitting meditation techniques, the variety of meditation with movement seems limitless. A comprehensive overview of meditation techniques using movement is still missing in literature. Thus, it seems very worthwhile to take a closer look at these techniques and disentangle their specificities and working mechanisms. Subsequently, researchers could compare movement-based meditation techniques to other basic techniques.

In the long run, all of these efforts could contribute to establishing one or more theories of meditation. This endeavor is not only imperative for the future of scientific research into meditation (Sedlmeier et al., 2014; Sedlmeier et al., 2016), but also highly promising in understanding the phenomenon of meditation (or the phenomena of different kinds of meditations) more thoroughly. With the proposed classification system, we hope to have taken an important step toward achieving this goal and encourage future scientific investigation into this matter.

## Conclusion

A broad range of diverse meditation techniques was effectively depicted in the novel classification system presented in this paper. This classification system is the first to be derived empirically by requesting experts’ evaluations. The dimensions depicted in our classification system shed new light on previous categorizations and shift the focus from cognitive to embodied variables of interest. We hope that our classification system will be useful for future studies and the development of one or more profound theories of meditation.

## Data Availability Statement

The datasets generated for this study are available on request to the corresponding author.

## Ethics Statement

The studies involving human participants were reviewed and approved by Ethikkommission, Fakultät für Human- und Sozialwissenschaften, Technische Universität Chemnitz. The patients/participants provided their written informed consent to participate in this study.

## Author Contributions

KM designed and executed the studies, analyzed the data, and wrote the first draft of the manuscript. PS conceptualized the studies and supervised the data analyses. Both authors worked on the final version of the manuscript.

## Conflict of Interest

The authors declare that the research was conducted in the absence of any commercial or financial relationships that could be construed as a potential conflict of interest.
